# Single-cell profiling reveals transcriptomic signatures of vascular endothelial cells in non-healing diabetic foot ulcers

**DOI:** 10.3389/fendo.2023.1275612

**Published:** 2023-12-01

**Authors:** Yangzhou Lu, Xiaogang Liu, Jingling Zhao, Fan Bie, Yiling Liu, Julin Xie, Peng Wang, Junyou Zhu, Yahui Xiong, Shitian Qin, Fan Yang, Lei Chen, Yingbin Xu

**Affiliations:** ^1^ Department of Burn, Wound Repair & Reconstruction, The First Affiliated Hospital of Sun Yat-Sen University, Guangzhou, Guangdong, China; ^2^ Guangdong Provincial Engineering Technology Research Center of Burn and Wound Accurate Diagnosis and Treatment Key Technology and Series of Products, Sun Yat-Sen University, Guangzhou, Guangdong, China; ^3^ Institute of Precision Medicine, The First Affiliated Hospital, Sun Yat-Sen University, Guangzhou, Guangdong, China

**Keywords:** single-cell RNA sequencing, diabetic foot ulcers, non-healing wounds, vascular endothelial cell, immune, inflammation, angiogenesis

## Abstract

**Background:**

The treatment of diabetic foot ulcers (DFUs) poses a challenging medical problem that has long plagued individuals with diabetes. Clinically, wounds that fail to heal for more than 12 weeks after the formation of DFUs are referred to as non-healing/chronic wounds. Among various factors contributing to the non-healing of DFUs, the impairment of skin microvascular endothelial cell function caused by high glucose plays a crucial role. Our study aimed to reveal the transcriptomic signatures of non-healing DFUs endothelial cells, providing novel intervention targets for treatment strategies.

**Methods:**

Based on the GEO dataset (GSE165816), we selected DFU-Healer, DFU-Non-healer, and healthy non-diabetic controls as research subjects. Single-cell RNA transcriptomic sequencing technology was employed to analyze the heterogeneity of endothelial cells in different skin tissue samples and identify healing-related endothelial cell subpopulations. Immunofluorescence was applied to validate the sequencing results on clinical specimens.

**Results:**

The number of endothelial cells and vascular density showed no significant differences among the three groups of skin specimens. However, endothelial cells from non-healing DFUs exhibited apparent inhibition of angiogenesis, inflammation, and immune-related signaling pathways. The expression of CCND1, ENO1, HIF1α, and SERPINE1 was significantly downregulated at the transcriptomic and histological levels. Further analysis demonstrated that healing-related endothelial cell subpopulations in non-healing DFUs has limited connection with other cell types and weaker differentiation ability.

**Conclusion:**

At the single-cell level, we uncovered the molecular and functional specificity of endothelial cells in non-healing DFUs and highlighted the importance of endothelial cell immune-mediated capability in angiogenesis and wound healing. This provides new insights for the treatment of DFUs.

## Introduction

According to the World Health Organization (WHO), diabetes is the third most serious chronic disease threatening human health, following cancer and cardiovascular diseases. Diabetic foot ulcer (DFU) is one of the most common complications among diabetic patients. Globally, it is estimated that 9.1 to 26.1 million people will develop DFU each year, with a lifetime incidence rate of 15-25% among diabetes patients (1). The healing of DFU involves complex physiological processes that include multiple cell types and cytokine involvement (2). In clinical practice, some DFUs heal well after active treatment, while others remain unhealed. Generally, DFU wounds that do not heal within 12 weeks are considered non-healing or chronic wounds (3, 4). The mechanisms underlying the different treatment outcomes of DFUs are not yet clear. However, it has been observed that diabetic patients with concomitant peripheral vascular diseases have the worst prognosis for foot ulceration (5). This may be due to biological and functional damage to endothelial cells (ECs) caused by factors such as high glucose and hypoxia (6–8). Among the many factors influencing the healing of DFUs, the function of endothelial cells is a crucial determinant of wound healing (9, 10). However, the transcriptomic signatures of endothelial cells in non-healing DFUs have been overlooked in existing research. Endothelial cells (ECs) not only form the inner lining of arteries, veins, and capillaries but also serve as endocrine cells that mediate immune and inflammatory responses. Different subtypes of ECs exhibit tissue-specific and vascular-type-specific immunoregulatory functions (11) and play a critical role in angiogenesis through various signaling pathways (12). Single-cell RNA sequencing (scRNA-seq) technology has become the most advanced method for revealing the heterogeneity and complexity of RNA transcripts within individual cells and for uncovering the composition and functions of different cell types in tissues, organs, and organisms (13). Georgios Theocharidis et al. (14) performed debridement surgery on DFU patients and collected skin samples from the wound site for research purposes. They defined DFU-Healer as patients whose wounds healed within 12 weeks after surgery and DFU-Non-healer as patients whose wounds remained non-healing. They analyzed the single-cell transcriptomic landscape and deposited the single-cell data in the Gene Expression Omnibus (GEO) database. Based on their GEO dataset (GSE165816), our study selected DFU-Healer, DFU-Non-healer, and healthy non-diabetic controls as research subjects to reveal differentially expressed genes and functional characteristics of endothelial cells that influence the healing of DFUs, providing reference for the clinical treatment of this disease.

## Methods

### Subjects

The research data were obtained from the NCBI Gene Expression Omnibus (GSE165816) dataset, comprising a total of 25 samples. Non-diabetic patients (n = 10) who underwent foot surgery for various reasons, such as corrective surgery for hallux valgus, were included as healthy controls. Diabetic foot ulcer patients (n = 11) underwent surgical excision of the ulcer, providing sufficient wound and peri-wound tissue for analysis. The DFU patients were followed up for 12 weeks post-surgery and were divided into two groups based on wound healing status: the ulcer healing group and the ulcer non-healing group (healers; n = 7, non-healers; n = 4). Participants with any diseases or medications that could potentially affect wound healing, other than diabetes, were excluded from the study (14). Additionally, skin samples from DFU patients (healers; n = 5, non-healers; n = 4) and non-DFU patients (control group; n = 3) were collected by our research team according to the above criteria for immunofluorescence staining. DFU patient specimens comprised ulcers and skin located 2-10mm away from the ulcer edge.All skin specimens consisted of full-thickness skin tissue, excluding the ulcer site, encompassing the epidermis, dermis, and subcutaneous tissue, with a volume ranging from 0.8 to 2.5 cm^3^. **Supplementary Dataset 1** includes clinical details of the participants in the study. There were no significant differences in the major biological characteristics among the groups.

### Data processing and analysis

The cell UMI (Unique Molecular Identifier) data table for each sample was directly downloaded from the GSE165816 dataset. The cell UMI data from the 25 samples used in this project were extracted. Cells entering the apoptosis program were filtered based on three criteria: gene expression counts between 500 and 5,000, UMI counts between 500 and 10,000, and mitochondrial gene expression percentage not exceeding 25%. Cells expressing multiple immune cell markers [T cell: CD8A, CD3D, CD3E; B cell: CD19; Macrophage: CD14, CD163; Dendritic: CD11c(ITG AX)] were filtered out. After cell filtering, the remaining valid cells were first normalized at the cell level. The NormalizeData tool of the Seurat package was used to normalize the gene expression levels of each cell, ensuring that the total expression of each cell summed up to 10,000. Next, the ScaleData tool was used to scale the cells based on the total UMI counts and mitochondrial gene expression levels, performing linear regression on the cell expression levels. The FindVariableFeatures tool was then employed to identify variable genes based on the average gene expression and gene expression variability of cells. The threshold for variable gene selection was an average gene expression between 0.125 and 3, and the top 2,000 variable genes were selected based on decreasing gene expression variability. The RunPCA tool of the Seurat package was used to perform PCA (Principal Component Analysis) on the cells based on the expression levels of variable genes. The top 18 principal components were selected, and t-SNE (t-Distributed Stochastic Neighbor Embedding) dimensionality reduction was applied to the cells using the FindCluster tool with a resolution of 0.5 (0.8 for subclustering of vascular endothelial cells) for cell clustering. Cell types were identified based on marker information provided in the referenced article (https://doi.org/10.1101/2021.03.11.434413). The FindMarkers tool of the Seurat package was used to analyze differential gene expression in the sample cells. The enrichKEGG function of the clusterProfiler package was employed to perform KEGG Pathway enrichment analysis on significantly differentially expressed genes, with the analysis threshold set at pvalueCutoff = 1, qvalueCutoff = 1, minGSSize = 1, and maxGSSize = 1000. Enrichment results with a p-value below 0.05 were considered significant.

### Differential gene analysis

Significantly differentially expressed transcription factor genes were identified based on the transcription factor information for the human species available in the Human Transcription Factor Database (HumanTFDB, http://bioinfo.life.hust.edu.cn/HumanTFDB#!/). Using the target gene data information of human transcription factors collected in the TRRUST database, a search was conducted for target genes of the transcription factors, and expressed target genes were filtered. The targeting relationships between transcription factors and target genes were listed. The enrichKEGG function in the clusterProfiler package was used to perform KEGG Pathway enrichment analysis on the target genes, specifically focusing on immune and inflammation-related pathways. The analysis thresholds were set as pvalueCutoff = 1, qvalueCutoff = 1, minGSSize = 1, and maxGSSize = 1000. Enrichment results with a p-value below 0.05 were considered significant. In the significantly differentially expressed gene enrichment pathways, immune and inflammation-related pathways were also selected. A Venn analysis was performed on the two sets of immune and inflammation-related pathways to obtain the common pathways. A Venn analysis was then conducted on the genes corresponding to the common pathways to identify the shared genes. The differential genes and target genes were analyzed for protein-protein interactions using the STRING protein interaction database (http://string-db.org/) for the human species, and the Cytoscape software was used for visualization.

### Specimen collection and sectioning

1. Specimen collection: Fresh tissue was fixed in 4% paraformaldehyde universal tissue fixativea (Biosharp, BL539A) for more than 24 hours. The tissue was taken out from the fixative solution and trimmed to the desired area using a surgical knife in a fume hood. The trimmed tissue was placed in a dehydration container along with the corresponding labels.
2. Dehydration and paraffin embedding: The dehydration container was placed in the biological tissue Dehydrator (ZEEDO, HS-569) for gradual ethanol dehydration. The sequence of dehydration was as follows: 75% ethanol for 4 hours, 85% ethanol for 2 hours, 90% ethanol for 2 hours, 95% ethanol for 1 hour, absolute ethanol I for 30 minutes, absolute ethanol II for 30 minutes, alcohol-benzene for 5-10 minutes, xylene I for 5-10 minutes, xylene II for 5-10 minutes, 65°C melting paraffin I for 1 hour, 65°C melting paraffin II for 1 hour, 65°C melting paraffin III for 1 hour.
3. Embedding: The dehydrated tissue was embedded using an embedding machine (ZEEDO, ES-300). First, the melted paraffin was placed in an embedding mold. Before the paraffin solidified, the tissue was taken out from the dehydration container, placed in the embedding mold according to the embedding surface requirements, and labeled accordingly. The embedding mold was cooled on a -20°C cold plate, and once the paraffin solidified, the paraffin block was removed from the embedding mold and trimmed.
4. Sectioning: The trimmed paraffin block was cooled on a -20°C cold plate, and then placed in the paraffin microtome (ZEEDO, HS-3345)for sectioning at a thickness of 4μm. The sections were floated on a water bath at 40°C to flatten the tissue, and the tissue was lifted onto glass slides. The slides were baked in a 60°C oven to dry and deparaffinize the sections. After deparaffinization and hydration, the slides were stored at room temperature for further use.

### Immunofluorescence staining and imaging

1. Dewaxing of paraffin sections: The sections were sequentially placed in dewaxing solution I (Eco-friendly) (Phygene, PH1900)for 10 minutes, dewaxing solution II for 10 minutes, dewaxing solution III for 10 minutes, absolute ethanol I for 5 minutes, absolute ethanol II for 5 minutes, absolute ethanol III for 5 minutes, and then rinsed with distilled water.
2. Antigen retrieval: In a transparent beaker, poured 500 mL of 50X sodium citrate antigen retrieval solution (Codow, CD434600), placed the tissue slides into the antigen retrieval solution, and put them together in a microwave oven. Heated them on high heat for 6-8 minutes. Carefully observed the heating of the retrieval solution and, once the solution boiled (to prevent excessive evaporation of the buffer and to avoid drying out the slides), turned off the heat, allowing it to cool down at room temperature.The slides were placed in PBS (pH 7.4) and washed on a decolorizing shaker for 3 times, 5 minutes each time.
3. Inactivation of endogenous hydrogen peroxidase: A circle was drawn around the tissue using a peroxidase-blocking pen. The slides were then placed in a 3% hydrogen peroxide solution and incubated at room temperature, protected from light, for 25 minutes to block endogenous peroxidase. After that, the slides were placed in PBS (pH 7.4) and washed on a decolorizing shaker for 3 times, 5 minutes each time.
4. Serum blocking: The PBS was removed and added 10% rabbit serum (Acmec, AC17053) for blocking for 30 minutes.
5. Primary antibody incubation: The blocking solution was removed and the prepared primary antibody was added. The slides were then placed flat in a humid chamber and incubated overnight at 4°C.
6. Secondary antibody/HRP inucubation: The slides were placed in PBS (pH 7.4) and washed on a decolorizing shaker for 3 times, 5 minutes each time. After excess liquid was gently shaken off, the corresponding HRP-labeled secondary antibody was added within the drawn circle and incubated at room temperature for 50 minutes.
7. Addition of TSA dye: The slides were placed in PBS (pH 7.4) and washed on a decolorizing shaker for 3 times, 5 minutes each time. After excess liquid was gently shaken off, the TSA reagent was added within the drawn circle and incubated at room temperature, protected from light, for 10 minutes. After incubation, the slides were placed in TBST and washed on a decolorizing shaker for 3 times, 5 minutes each time.
8. Antigen retrieval: The washed slides were subjected to the same procedure as described in step 2.
9. Second round of antibody incubation: Steps 4-7 were repeated. In step 5, the second primary antibody was applied, and in step 7, the second TSA dye was used.
10. DAPI counterstaining of cell nuclei: After excess liquid was gently shaken off, DAPI staining solution was added within the drawn circle and incubated at room temperature, protected from light, for 10 minutes.
11. Quenching of autofluorescence: The slides were placed in PBS (pH 7.4) and washed on a decolorizing shaker for 3 times, 5 minutes each time. After excess liquid was gently shaken off, autofluorescence quenching reagent B (Servicebio, G1221-2)was added within the drawn circle, incubated for 5 minutes, and then rinsed with running water for 10 minutes.
12. Mounting: The slides were mounted with anti-fluorescence quenching mounting medium (Servicebio, G1401).
13. Image acquisition: The upright fluorescence microscope (Nikon, Eclipse C1) was using for image acquisition. DAPI excitation wavelength 330-380 nm, emission wavelength 420 nm; SPGreen (FITC) excitation wavelength 465-495 nm, emission wavelength 515-555 nm; SPOrange (CY3) excitation wavelength 510-560 nm, emission wavelength 590 nm.

Note: Information regarding the antibodies and fluorescent dyes used in immunofluorescence staining is provided in the **Table 1**:

**Table 1:** Serum sPD-L1 changes before and after 3-4 cycles of PD-1 inhibitors treatment in advanced non-small cell lung cancer (NSCLC) patients.

Name	CD31	CCND1	ENO1	HIF1α	SERPINE1	Goat Anti-Rabbit IgG (H+L) HRP	FITC-Tyramide(TSA)	CY3-Tyramide (TSA)	DAPI
Company	Affinity Biosciences	Affinity Biosciences	Affinity Biosciences	Affinity Biosciences	Affinity Biosciences	Affinity Biosciences	Servicebio	Servicebio	Abcam
Catalog Number	AF6191	AF0931	DF6191	AF1009	AF5176	S0001	G1222	G1223	ab228549
Dilution	1:200	1:200	1:100	1:200	1:100	1:200	1:500	1:500	1:10000

## Results

### Identification and gene features of skin tissue cells

**Figure 1:**
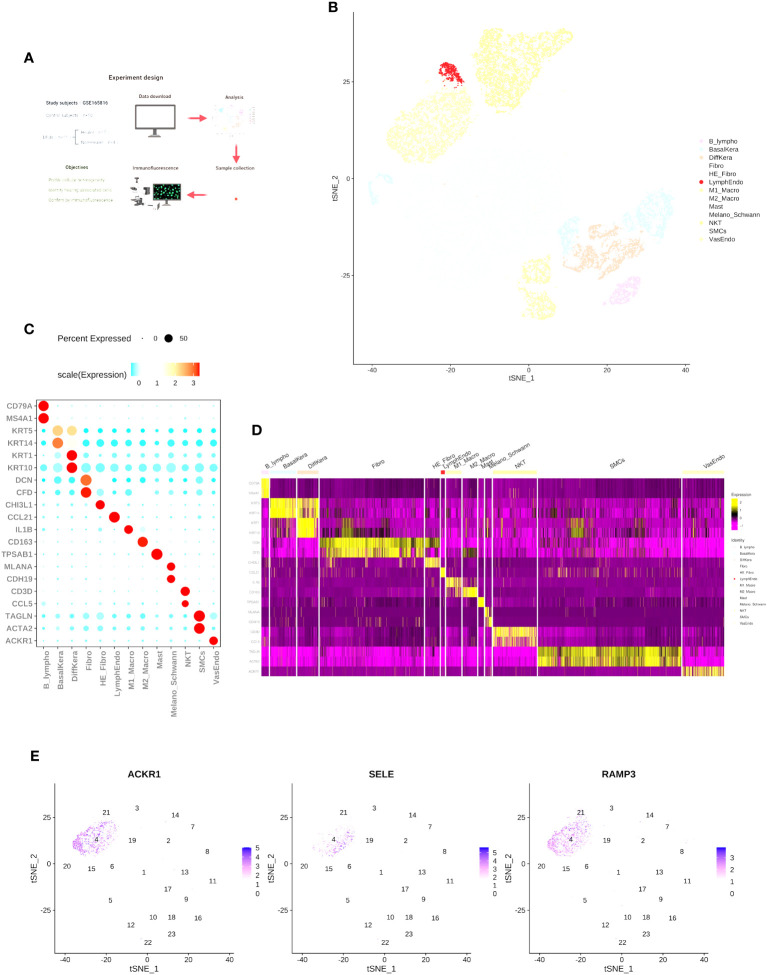
Single-cell RNA-seq reveals heterogeneity in normal skin and diabetic foot ulcers. **(A)** Overview of the study design and the number of samples in each clinical group. **(B)** t-SNE plot showing the composition of the entire dataset consisting of 53,199 cells. Cells are color-coded by orthogonal-generated clusters and labeled based on manual cell type annotations (HE-Fibro, Healing-enriched fibroblasts; Fibro, Fibroblasts; SMCs, Smooth muscle cells; BasalKera, Basal keratinocytes; DiffKera, Differentiated keratinocytes; Melano/Schwann, Melanocytes and Schwann cells; Mast, Mast cells; VasEndo, Vascular endothelial cells; M1-macro, M1 macrophages; M2-macro, M2 macrophages; NKT, NK cells and T lymphocytes; LymphEndo, Lymphatic endothelial cells; B-lympho, B lymphocytes). **(C)** Dot plots displaying the expression of cell type-specific marker genes used for cell type annotation. The size of the dots represents the percentage of cells in each cell cluster expressing the marker gene, and the color represents the average proportion of expression levels (blue: low, red: high). **(D)** Heatmap showing the top highly expressed genes in each cell cluster. **(E)** Expression profiles of characteristic genes for vascular endothelial cells: (I) ACKR1, (II) SELE, and (III) RAMP3. The schematic diagram in **(A)** was created using BioRender (BioRender.com).

To determine the cell types and gene features of normal skin and DFU skin cells, we referred to the study by Georgios Theocharidis et al. (14) and downloaded a portion of the raw dataset (GSE165816) for single-cell expression analysis **Supplementary Dataset 2**. We analyzed a total of 25 samples from 11 diabetes patients (7 DFU-Healers and 4 DFU-Non-Healers) and 10 healthy non-diabetic subjects. The study groups, objectives, and analysis strategy are shown in **Figure 1A**. In summary, following the cell type marker information provided in the research of Georgios Theocharidis et al. (14), we analyzed 53,199 cells (24,922 from normal skin, 12,173 from DFU-Healers, and 17,743 from DFU-Non-Healers) and created a gene expression matrix for each cell. We used t-SNE plot and graph-based clustering for dimensionality reduction, resulting in the identification of 13 distinct cell types (**Figure 1B**). We identified most of the typical cell types observed in human skin (15, 16), including smooth muscle cells (SMCs) (TAGLN+, ACTA2+), fibroblasts (Fibro) (DCN+, CFD+), HE-fibro (DCN+, CHI3L1+), vascular endothelial cells (VasEndo) (ACKR1+), differentiated keratinocytes (DiffKera) (KRT1+, KRT10+), basal keratinocytes (BasalKera) (KRT5+, KRT14+), NK and T cells (NKT) (CD3D+, CCL5+), M1 macrophages (M1-macro) (IL1B+), M2 macrophages (M2-macro) (CD163+), melanocytes and Schwann cells (Melano/Schwann) (MLANA+, CDH19+), lymphatic endothelial cells (LymphEndo) (CCL21+), B lymphocytes (B lymphos) (CD79A+, MS4A1+), and mast cells (TPSAB1+) (**Figures 1C, D**, **Supplementary Dataset 3**). The distribution of the main markers for vascular endothelial cells is shown in the t-SNE plot (**Figure 1E**).

### Exploring cellular heterogeneity in DFU-Healer, DFU-Non-Healer and healthy controls

**Figure 2:**
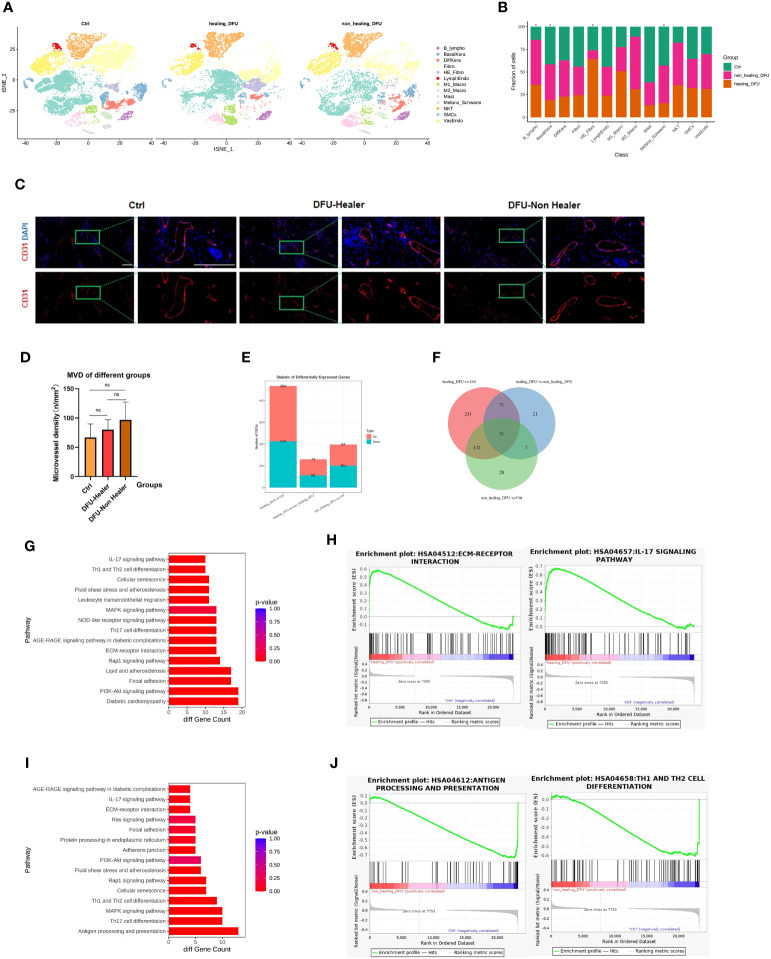
Single-cell transcriptomic analysis profiles comparing Healthy Control, DFU-Healer, and DFU-Non-Healer groups, describing gene features and healing-related biological pathways. **(A)** Separated t-SNE plot of the Healthy Control, DFU-Healer, and DFU-Non-Healer groups. Cell clusters were manually annotated based on the expression of specific markers, representing various known and novel cell types (as shown in **Figures 1C, D**). **(B)** Stacked bar plots showing the proportions of different cell types in the three clinical groups. Green: Healthy Control, Orange: DFU-Healer, Red: DFU-Non-Healer. Cell types with significant differences among the clinical groups are marked with asterisks. **(C)** Immunofluorescence staining showing the distribution of blood vessels in the three groups (healers; n = 5, non-healers; n = 4, healthy controls; n=3). CD31: Red, DAPI: Blue. Scale bars are 200 μm. **(D)** Bar graph displaying the statistical analysis of blood vessel density in the three groups (healers; n = 5, non-healers; n = 4, healthy controls; n=3). * denotes significant differences (p<0.05), ns denotes no significant difference (one- way ANOVA with Fisher’s LSD *post-hoc*). **(E)** Bar plots comparing the number of differentially expressed genes in endothelial cells among the three clinical groups in pairwise fashion. **(F)** Venn diagram showing the number of commonly significant differentially expressed genes among the three clinical groups. **(G)** Immune and inflammation related KEGG pathways for significantly differentially expressed genes of vascular endothelial cells between DFU-Healer and Healthy Control. **(H)** The significantly enriched GESA plot(FDR<0.05) based on the KEGG pathways in **(G)**. **(I)** Immune and inflammation related KEGG pathways for significantly differentially expressed genes of vascular endothelial cells between DFU-Non-Healer and Healthy Control. **(J)** The significantly enriched GESA plot(FDR<0.05) based on the KEGG pathways in **(I)**.

To assess the cellular heterogeneity, gene expression, and molecular pathway changes among different clinical groups, we generated separate t-SNE plots based on samples from normal skin, DFU-Healer, and DFU-Non-Healer (**Figure 2A**). The results of cell abundance analysis for the three groups (**Figure 2B**) showed significant differences (p<0.05, **Supplementary Figure 1**) in B-lymphocytes, HE-Fibroblasts, Basal keratinocytes, lymphatic endothelial cells, and melanocytes/Schwann cells among the clinical groups. Specifically, B-lymphocytes were significantly higher in DFU-Healer compared to the Healthy Control group, suggesting the enrichment of B-lymphocytes following wound formation, which may be associated with increased collagen deposition and maturation, enhanced angiogenesis, and promoted nerve growth (17). Notably, B lymphocytes in DFU-Non-Healer were also significantly elevated comparing to Healthy Controls. The underlying reason could be attributed to the varying roles of different B cell subpopulations in wound healing, which warrants further research to elucidate the mechanisms involved (18). In DFU-Healer, the abundance of HE-Fibroblasts was 64.11 ± 2.06% (mean ± SE), and M1 macrophages were 50.79 ± 1.59%, while in DFU-Non-Healer, HE-Fibroblasts were 9.84 ± 0.50%, and M1 macrophages were 26.58 ± 1.16%. In the healthy Ctrl group, HE-Fibroblasts were 26.04 ± 2.19%, and M1 macrophages were 22.62 ± 0.73%. The proportion of VasEndo was almost the same among the three groups (**Supplementary Dataset 4**). The paraffin sections of a total of 12 clinical samples from the three groups were subjected to immunofluorescence staining,with CD31 used as the marker for endothelial cells (**Figure 2C**). Fluorescence microscopy was employed for observation. Any individual endothelial cell or endothelial-cell cluster stained by the CD31 antibody, regardless of whether they formed luminal structures, as long as they had clear boundaries with surrounding blood vessels, were considered countable vessels (19). The most densely vascularized areas were observed at 10× magnification, and three random subregions within these areas were selected. At 40× magnification (grid area 0.1 mm^2^), photographs of these three subregions were taken, and vessel numbers were counted individually. The average value of vessel numbers was calculated for each specimen, and vascular density was expressed as vessels per square millimeter (n/mm^2^). Finally, one-way ANOVA statistical analysis (**Figure 2D**) was performed to compare vascular density among the three groups, revealing no significant differences between them. The comparison of the number of significantly differentially expressed genes among different groups illustrates the upregulation or downregulation of genes (**Figure 2E**, **Supplementary Dataset 5**). And there were 31 commonly significant differentially expressed genes among the three groups (**Figure 2F**). In DFU-Healer vs healthy Ctrls, 254 genes were upregulated, and 213 genes were downregulated. In DFU-Healer vs DFU-Non-Healer, 74 genes were upregulated, and 56 genes were downregulated. In DFU-Non-Healer vs healthy Ctrls, 97 genes were upregulated, and 101 genes were downregulated. To compare the vascular endothelial cells differentially expressed genes more detailedly between DFU-Healer and healthy Ctrls, KEGG analysis was performed on their transcriptome profiles (**Figure 2G**). It was found that vascular endothelial cells exhibited upregulation in several pathways associated with ECM receptor signaling and inflammation, as shown by the heatmap of corresponding gene expression (**Supplementary Figure 2**). GSEA enrichment analysis revealed significant activation of the ECM-receptor interaction and IL-17 signaling pathway in DFU-Healer (FDR<0.05) (**Figure 2H**), which may be related to the appropriate inflammatory response mediated by vascular endothelial cells after wound formation, promoting angiogenesis and wound healing (20, 21). Further KEGG analysis of the differentially expressed genes between DFU-Non-Healer and healthy controls revealed downregulation of several pathways associated with immune response in vascular endothelial cells (**Figure 2I**), as shown by the heatmap of corresponding gene expression (**Supplementary Figure 3**). GSEA enrichment analysis showed significant inhibition of antigen processing and presentation, Th17 cell differentiation, and Th1 and Th2 cell differentiation in DFU-Non Healer (**Figure 2J**, **Supplementary Figure 4**). The failure of endothelial cells in DFU-Non-Healer to exert immune regulatory functions similar to T cells mediating inflammatory response after wound formation, may affecting wound angiogenesis and healing (11, 22, 23).

### Comparative analysis of vascular endothelial cells revealed downregulation of key healing-related gene expression in DFU-Non-Healer

**Figure 3:**
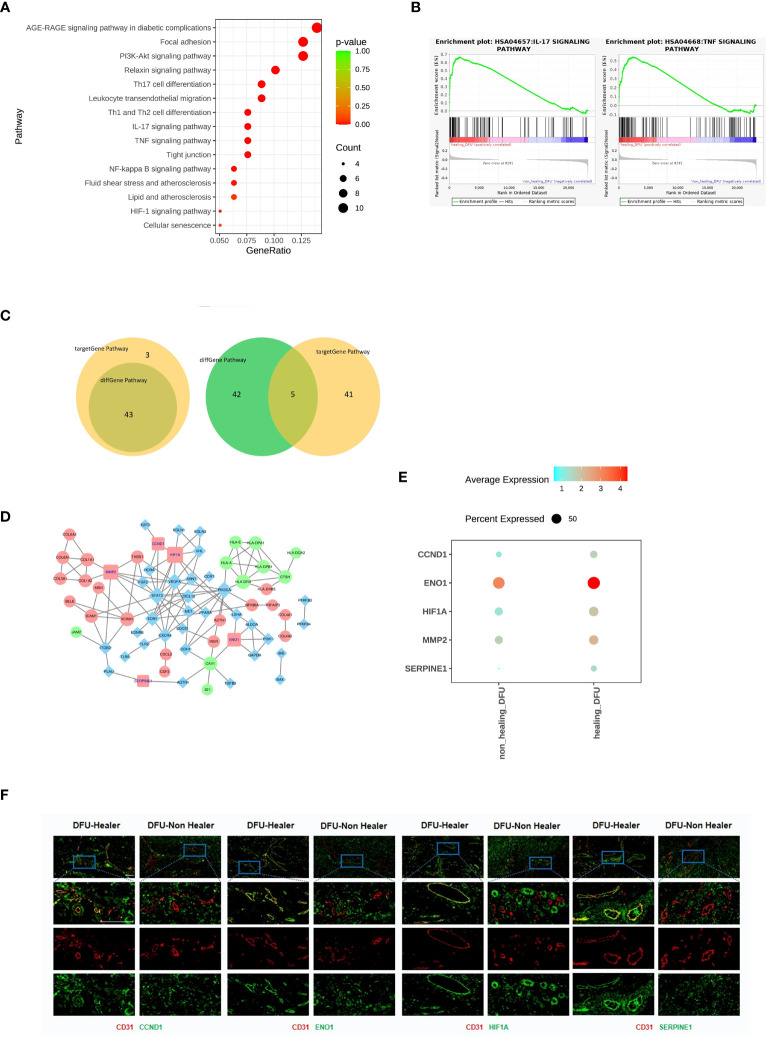
Comparison of the gene characteristics, healing-related biological pathways, and significantly differentially expressed genes between the DFU-Healer and DFU-Non-Healer groups. **(A)** Bubble plot illustrating the significant differences in immune and inflammation related KEGG pathway between DFU-Healer and DFU-Non-Healer endothelial cells. **(B)** The significantly enriched GESA plot(FDR<0.05) based on the KEGG pathways in **(A)**. **(C)** The Venn diagram showcases the intersection of 46 immune and inflammation related pathways enriched with significantly differentially expressed genes in DFU-Healer and DFU-Non Healer endothelial cells, as well as the 43 pathways enriched with immune and inflammation related target genes. The diagram reveals a total of 43 common pathways (left), where the DFU-Healer and DFU-Non-Healer endothelial cells share 46 healing-related genes and 47 target genes. The Venn diagram displays 5 genes that are common to both groups (right): CCND1, ENO1, HIF1α, MMP2, and SERPINE1. **(D)** The Protein-Protein Interaction Network analysis demonstrates the interactions among these 5 common genes in **(C)**. **(E)** Dot plots illustrates the differential expression (p<0.05) of CCND1, ENO1, HIF1α, MMP2, and SERPINE1 between the DFU-Healer and DFU-Non Healer groups. **(F)** The immunofluorescence validation of protein expression, with CD31 labeled in red, CCND1, ENO1, HIF1α, and SERPINE1 labeled in green, and DAPI staining in blue. Scale bars are 200 μm.

The differentially expressed gene analysis in DFU-Healer and DFU-Non-Healer showed enrichment of immune and inflammation related pathways, suggesting that vascular endothelial cells mediate immune regulation and inflammatory responses (**Figure 3A**), as shown by the heatmap of corresponding gene expression (**Supplementary Figure 5**). To further compare the gene expression profiles of vascular endothelial cells between DFU-Healer and DFU-Non-Healer, we performed GSEA on their transcriptomic profiles. We found significant enrichment of eight KEGG signaling pathways in DFU-Healer, including the AGE-RAGE signaling pathway in diabetic complications, Focal adhesion, PI3K-Akt signaling pathway, Relaxin signaling pathway, IL-17 signaling pathway, TNF signaling pathway, NF-kappa B signaling pathway, and HIF-1 signaling pathway. All these pathways are associated with immune, inflammation, and vascularization (24) (**Figure 3B**, **Supplementary Figure 6**). This suggests that vascular endothelial cells in the DFU-Non-Healer group have weaker immune regulation, inflammatory response, and vascular generation potential compared to the DFU-Healer group.

There were 130 significantly differentially expressed genes between DFU-Healer and DFU-Non-Healer (**Figure 2E**), which were enriched in 193 pathways, including 46 immune and inflammation related pathways. Using the Human Transcription Factor Database (HumanTFDB), we identified two significantly differentially expressed transcription factors, HIF1α and ID1. Searching the TRRUST database for the corresponding target genes of HIF-1α and ID1, we found 77 target genes. Transcription factors and their target genes exhibit a one-to-many relationship (**Supplementary Dataset 6**). We performed KEGG Pathway enrichment analysis on these 77 target genes and identified a total of 208 KEGG pathways, of which 88 pathways were significantly enriched including 43 immune and inflammation related pathways. The top 30 significantly enriched pathways among the 88 pathways were selected for scatter plot display (**Supplementary Figure 7**). Out of the immune and inflammation related pathways enriched in significantly differentially expressed genes and target genes, 43 were exhibited a common intersection. (**Figure 3C**, left, **Supplementary Dataset 7**). Among these 43 shared pathways, there were a total of 46 significantly differentially expressed genes and 47 transcription factor target genes. Analysis of the relationship between these two gene sets revealed that five genes (CCND1, ENO1, HIF1α, MMP2, SERPINE1) were common (**Figure 3C**, right). Cyclin D1 (CyD1) is a key cell cycle regulatory molecule with immunoregulatory functions. It is significantly upregulated at the site of inflammation and its synergistic interaction with VEGFA promotes angiogenesis and vascular permeability (25–27). Enolase is a glycolytic enzyme that catalyzes the interconversion of 2-phosphoglycerate and phosphoenolpyruvate. Rheumatoid arthritis (RA) patients have increased surface expression of enolase-1 (ENO1) on their immune cells, leading to enhanced inflammatory response and promoting tumor angiogenesis (28–30). Hypoxia-inducible factor 1-alpha (HIF1α) is one of the major regulatory factors involved in cellular responses to hypoxia. It plays a role in regulating cell metabolism and immune cell effector function. HIF1α is a key metabolic reprogrammer that promotes the expression of inflammatory genes in inflammatory cells. Overexpression of HIF-1α promotes invasion, migration, proliferation, and tubule formation ability of endothelial cells, and it has a role in promoting tissue angiogenesis and diabetic foot ulcer healing (31–35). Matrix metalloproteinase-2 (MMP2) is one of the members of the matrix metalloproteinase gene family (MMPs) and is highly expressed in neuroinflammation. Inhibiting MMP2 expression can suppress inflammatory pathways and angiogenesis (36–38). Plasminogen activator inhibitor-1 (PAI-1, SERPINE1) is a major inhibitor of tissue plasminogen activator and is associated with tumor progression and angiogenesis. Downregulation of SERPINE1 expression in ECs can inhibit vascular formation. In cell experiments, SERPINE1 directly inhibits eNOS activity, reduces NO synthesis, and enhances endothelial cell function (39–41). Protein-Protein Interaction Network analysis using the STRING protein interaction database and Cytoscape software was performed on the 46 significantly differentially expressed genes and 47 target genes (**Figure 3D**). CCND1, ENO1, HIF1α, MMP2, and SERPINE1 showed significantly lower expression in DFU-Non-Healer compared to DFU-Healer (p<0.05) (**Figure 3E**). Immunofluorescence staining of CD31 in conjunction with CCND1, ENO1, HIF1α, MMP2, and SERPINE1 showed co-expression of CD31 with CCND1, ENO1, HIF1α, and SERPINE1 on the vascular wall in the DFU-Healer group, while no significant co-expression was observed in the DFU-Non-Healer group (**Figure 3F**). This suggests that the high expression of CCND1, ENO1, HIF1α, and SERPINE1 is beneficial for wound healing, while low expression may lead to non-healing wounds.It is worth noting that MMP2 was not significantly expressed in the vascular walls of both sample groups, which may be related to post-transcriptional regulation, translation, and protein degradation of mRNA (42–44).

### Exploration of healing-related subpopulations in endothelial cells

**Figure 4:**
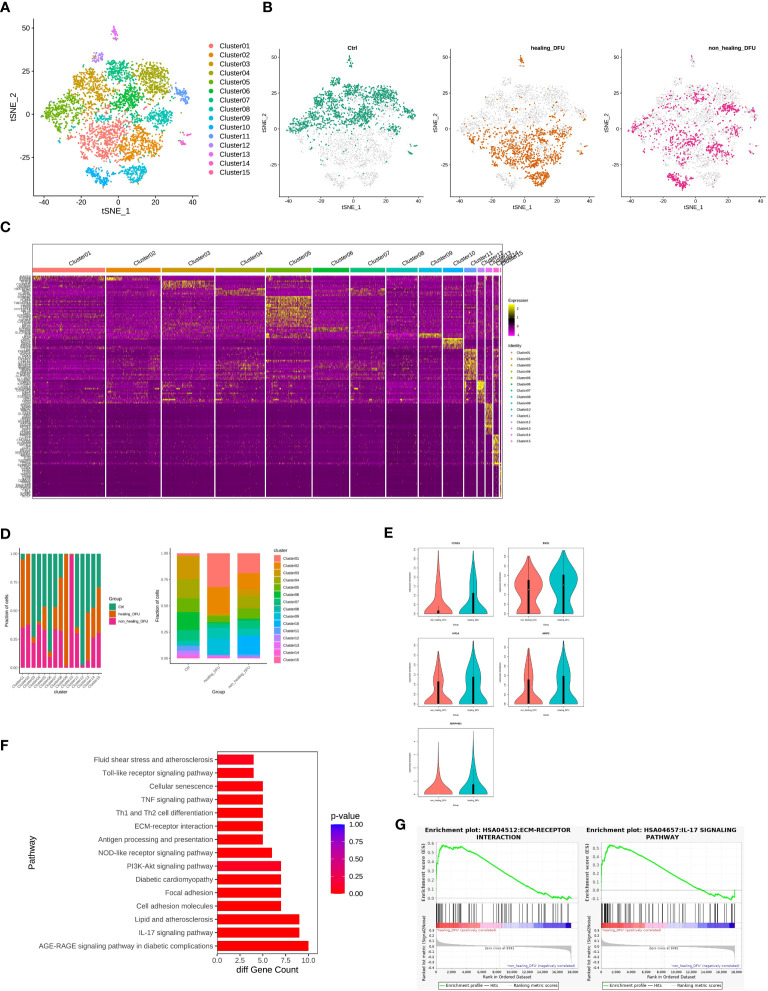
Comparison of gene features and healing-related biological pathways in subpopulations of endothelial cells between DFU-Healer and DFU-Non-Healer groups. **(A)** t-SNE plot embedding of the entire dataset comprising 4948 cells. Cells are colored by orthogonal-generated clusters and labeled based on manual cell type annotations. **(B)** t-SNE plot showing the separation of subpopulations of endothelial cells from Healthy Control, DFU-Healer, and DFU-Non-Healer groups. **(C)** Heatmap displaying top highly expressed genes within each cell subpopulation. **(D)** Bar plot depicting the proportions of endothelial cell subpopulations. **(E)** Violin plot illustrating the differential expression of CCND1, ENO1, HIF1α, MMP2, and SERPINE1 between healing-related subpopulations 1, 2, and 5 (HE-VasEndo) in the DFU-Healer and DFU-Non-Healer groups. **(F)** KEGG pathway enrichment analysis of significantly differentially expressed genes in healing-related subpopulations 1, 2, and 5 (HE-VasEndo) of DFU-Healer and DFU-Non-Healer. **(G)** GSEA-KEGG showing significantly enriched pathways between DFU-Healer and DFU-Non-Healer.

We performed secondary subpopulation classification of endothelial cells, analyzing a total of 4948 cells (2136 from Healthy Control, 1578 from DFU-Healer, and 1234 from DFU-Non-Healer). A gene expression matrix was created for each cell, and t-SNE dimensionality reduction was performed. Using the FindCluster tool in the Seurat package with a resolution of 0.8, cells were clustered into 15 distinct cell types (**Figure 4A**). An independent t-SNE plot revealed different distributions of endothelial cell subpopulations among the three groups, indicating significant heterogeneity between the samples (**Figure 4B**). FindMarkers tool was used to analyze differentially expressed marker genes for each cell cluster relative to other clusters, and the results were visualized using a heatmap (**Figure 4C**). Comparative analysis of cell type abundance demonstrated differences in endothelial subclusters among the different clinical groups (**Figure 4D**). Specifically, Cluster 1 and Cluster 2 were the main subcluster types in the DFU-Healer group, with higher proportions compared to the other two groups. When analyzing Subclusters 1, 2, and 5 as a whole, we found that the expression of CCND1, ENO1, HIF1α, MMP2, and SERPINE1 was lower in the DFU-Non-Healer group compared to the DFU-Healer group (**Figure 4E**), with ENO1 and SERPINE1 showing significant downregulation (p<0.05). We defined Subclusters 1, 2, and 5 as Healing Enriched Vascular endothelial cell (HE-VasEndo). Further KEGG pathway enrichment analysis of significantly differentially expressed genes between the two groups identified a total of 47 enriched KEGG pathways, predominantly related to inflammation and immunity (**Figure 4F**). GSEA-KEGG analysis revealed that compared to DFU-Non-Healer, most of the inflammation, immunity, and extracellular matrix pathways were enriched in DFU-Healer, with significant enrichment observed in the AGE-RAGE signaling pathway in diabetic complications, IL-17 signaling pathway (45), Focal adhesion (46), PI3K-Akt signaling pathway (47), and ECM-receptor interaction (20) (**Figure 4G**), all of which are associated with angiogenesis. This suggests that HE-VasEndo plays a crucial role in promoting angiogenesis and wound healing and the absence of this endothelial subpopulation may contribute to non-healing wounds.

### Cell communication analysis revealing the intercellular connections and cell differentiation trajectories of HE-VasEndo, macrophages, smooth muscle cells (SMC), and HE-fibroblasts

**Figure 5:**
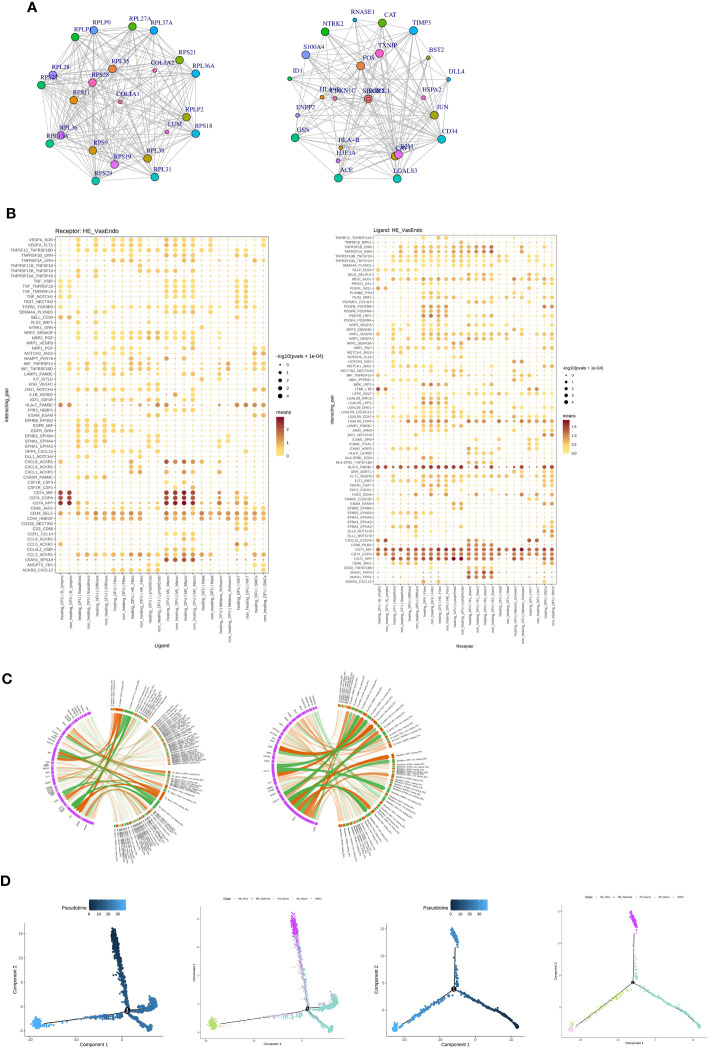
Analysis of potential ligand-receptor interactions and cellular state changes in the healing-related subpopulations of vascular endothelial cells (HE-VasEndo). **(A)** The interaction network diagrams illustrate the significantly upregulated (left) and downregulated (right) cytokine interactions in the enriched HE-VasEndo involved in healing. **(B)** The ligand-receptor pairs show significant and specific changes between different cell types and HE-VasEndo in the DFU-Healer and DFU-Non-Healer groups. The left panel displays the expression of receptors in HE-VasEndo, receiving ligand signals from other cell types. The right panel shows the expression of receptors in other cell types, receiving ligand signals from HE-VasEndo. **(C)** The circular plot demonstrates the associations between the receptors expressed in HE-VasEndo and the ligands expressed in B lymphocytes, keratinocytes and M1-Macro (left), as well as the ligands expressed in HE-VasEndo and the receptors expressed in B lymphocytes and keratinocytes (right). **(D)** The differentiation trajectory plots of cells associated with DFU-Healer and DFU-Non-Healer are shown. The blue panel represents the inferred evolution time of cells, while the colorful panel displays the evolutionary trajectories labeled with different cell type origins, with different colors corresponding to different cell subpopulations.

In comparison to DFU-Healer, DFU-Non-Healer showed significant downregulation of 74 genes and upregulation of 54 genes. The interactions among downregulated genes and upregulated genes were separately analyzed, resulting in 602 and 201 pairs of gene interactions, respectively. The top 25 genes with scores above 900 and the highest number of interactions were selected from the downregulated gene interactions to construct an interaction network diagram (**Figure 5A**, left). Similarly, the top 25 genes with the most interactions were selected from the upregulated gene interactions to construct another interaction network diagram (**Figure 5A**, right).

To compare the intercellular interactions between the two sample groups, we utilized the CellPhoneDB software to perform cell communication analysis across all cell populations. HE-VasEndo was considered as the receptor, while other cells served as ligands. A total of 1,172 ligand-receptor relationships were identified. From these, we selected 66 ligand-receptor pairs that met the criteria of having at least one significant relationship among 12 pairs and an average ligand-receptor score greater than 0.5 in the DFU-Non-Healer samples, or at least one significant relationship among 12 pairs and an average ligand-receptor score greater than 0.5 in the DFU-Healer samples (**Figure 5B**, left). The analysis of the effects of different cell types on HE-VasEndo revealed that B lymphocytes, M1 macrophages, and M2 macrophages had the strongest impact. Furthermore, compared to DFU-Healer, DFU-Non-Healer exhibited a relative deficiency in the effects of B lymphocytes, keratinocytes, and M1 macrophages on HE-VasEndo. The key differentially expressed ligands included CXCL8, CD44, and CCL5 for B lymphocytes, VEGFA, TNFRSF10A/B, SEMA4A, PLD2, and NRP2 for keratinocytes, and CXCL1, HLA-C, and NAMPT for M1 macrophages (**Figure 5C**, left). Additionally, when HE-VasEndo was considered as the ligand and other cells as the receptor, a total of 1,145 ligand-receptor relationships were analyzed, resulting in 76 ligand-receptor pairs (**Figure 5B**, right). Compared to DFU-Healer, DFU-Non-Healer exhibited a relative deficiency in the effects of HE-VasEndo on B lymphocytes and keratinocytes. The key differentially expressed ligands included FAM3C, FLT1, LGALS9, MIF, NRP1, and SELE for B lymphocytes, and NRP1/2, NOTCH1, LGALS9, and FLT1 for keratinocytes (**Figure 5C**, right).

To measure the transcriptional dynamics of the cell types of interest, we used Monocle (version: 2) to construct and compare the differentiation trajectories of HE-VasEndo, macrophages, SMC, and HE-fibroblasts between DFU-Healer and DFU-Non-Healer. From an evolutionary perspective of cell types analysis, the cell profiling results of the DFU-Healer suggested that HE-VasEndo cells are situated at an initial evolutionary position, followed by SMC and HE-Fibro cells, and eventually giving rise to M1-Micro and M2-Micro cells (**Figure 5D**, left; **Supplementary Figure 8**, above). However, the cell profiling results of the DFU-Non-Healer exhibited notable differences: SMC serves as the initial cell type, followed by HE-Fibro cells, with subsequent differentiation into two distinct pathways. One pathway leads to the formation of HE-VasEndo cells, while the other leads to the development of M1-Micro and M2-Micro cells (**Figure 5D**, right; **Supplementary Figure 8**, below). These results indicate that HE-VasEndo in DFU-Non-Healer represents a late-stage differentiated cell type with lower differentiation potential compared to other cells. HE-VasEndo in DFU-Healer represents an early-stage differentiated cell type with greater differentiation potential. It exhibits more stem cell-like characteristics and plays a positive role in vascular regeneration and wound healing (48, 49).

## Discussion

The normal process of wound healing involves inflammation, angiogenesis, and extracellular matrix (ECM) remodeling. The cellular players involved in healing include vascular endothelial cells, fibroblasts, keratinocytes, monocyte macrophages, neutrophils, lymphocytes, and other immune cells. Cytokines such as transforming growth factor(TGF)-β1, vascular endothelial growth factor (VEGF), soluble vascular cell adhesion molecule-1 (VCAM-1), platelet-derived growth factor (PDGF), and epidermal growth factor (EGF) influence wound healing (50–56). Among these, angiogenesis is crucial for wound healing, with vascular endothelial cells being the key participants (57). They actively control the dilation and constriction of blood vessels, as well as the extravasation of solutes, fluids, macromolecules, and hormones, including platelets and blood cells. They also guide inflammatory cells outside the blood vessels to areas requiring repair or defense against infection. Furthermore, endothelial cells play important roles in controlling blood flow, platelet adhesion and aggregation, leukocyte activation, adhesion, and translocation. They are closely involved in maintaining the balance between coagulation and fibrinolysis and play significant roles in regulating immune responses, inflammation, and angiogenesis (58).

Currently, the treatment strategies for DFUs include comprehensive approaches such as wound debridement, ulcer offloading, medication, and wound dressings (59). However, the management of non-healing DFUs remains a challenging clinical problem, causing significant psychological and economic burdens to individuals and consuming substantial healthcare resources (60). In the context of high blood glucose levels, reduced angiogenic factors, endothelial dysfunction, and vascular lumen narrowing impair vascularization of diabetic wounds, hindering wound healing (61). To the best of our knowledge, many studies have focused on alterations in the skin microenvironment of diabetic patients with diabetic mellitus (DM) or diabetic foot ulcers (DFUs), but there is limited research on vascular endothelial cells in recalcitrant DFUs. In this study, based on the original experimental data (GSE165816) from our previous research, we focused on the skin of healing and non-healing DFU patients, with healthy non-diabetic subjects’ skin serving as a control. This study provides the first insights into the transcriptomic signatures of vascular endothelial cells that influence vascularization and healing of diabetic wounds, laying the foundation for investigating the molecular mechanisms of non-healing vascular endothelial cells in DFUs.

The immune microenvironment of wound healing, including the proper activation, regulation, and distribution of various immune cells, is crucial for angiogenesis and healing. The process of wound angiogenesis involves the interplay between endothelial cells and the immune system. It is an integral part of both acute and chronic inflammation and is implicated in most immune-mediated diseases. In chronic inflammatory diseases, macrophages and lymphocytes infiltrate, tissue damage and repair occur simultaneously, and newly formed vessels become permanent. Angiogenesis and the inflammatory response are interdependent (62). By comparing the DFU-Healer and DFU-Non-Healer sample groups, the GSEA-KEGG pathway analysis results suggest that the inhibition of immune and inflammation related pathways in vascular endothelial cells of DFU-Non-Healer may impede vascularization and healing of DFUs. Therefore, it is evident that excessive suppression of local inflammatory responses in chronic non-healing DFU is detrimental to wound healing, while systemic suppression of inflammation is beneficial (14). Immune modulation is not exclusive to immune cells but also a characteristic of endothelial cells. The immune properties of vascular endothelial cells can mediate the wound microenvironment and maintain vascular function (11). Increasing evidence indicates that proper immune regulation and inflammation response can promote wound angiogenesis and healing, whereas excessive or dysregulated inflammatory responses lead to delayed wound healing (63, 64).

Extracellular matrix (ECM) plays a crucial role in various aspects of vascular biology. During the initiation of angiogenesis, ECM is involved in key signaling events that support the regulation of endothelial cell (EC) migration, invasion, proliferation, and survival. Moreover, temporary ECM acts as a flexible scaffold, establishing mechanical guidance between distant ECs and providing tissue cues in the absence of cell-cell contact. Lastly, through specific integrin-dependent signaling pathways, ECM controls the coordination of endothelial cell cytoskeleton to facilitate the complex process of vascular morphogenesis, wherein proliferating ECs organize into multicellular tubes with functional lumens. Therefore, the composition of ECM and its regulation of ECM degradation and remodeling play critical roles in controlling lumen and tube formation, as well as the ultimate stability and maturation of new blood vessels (65). In our study, we found that the inhibition of ECM receptor-related pathways in vascular endothelial cells of DFU-Non-Healer hinders vascularization and healing of diabetic foot ulcers.

Using single-cell transcriptomic sequencing analysis, our study revealed that five genes, CCND1, ENO1, HIF1α, MMP2, and SERPINE1, were significantly downregulated in DFU-Non-Healer compared to DFU-Healer. The differential expression of CCND1, ENO1, HIF1α, and SERPINE1 was further validated through immunofluorescence methods. These findings suggest that these genes play unique roles in promoting the healing of diabetic foot ulcers, and their deficiency impedes wound healing. However, the specific mechanisms need further verification through *in vitro* cell experiments. It is worth noting that single-cell sequencing showed no significant difference in the number of endothelial cells between the DFU-Healer and DFU-Non-Healer groups, and immunofluorescence revealed no significant difference in vascular density. This suggests that the healing of diabetic foot ulcers may be associated with the immune-mediated capacity of endothelial cells and vascular function, rather than cell quantity and vascular density (66, 67). Future longitudinal studies comparing DFU samples collected from the same patients at multiple time points during the wound healing process can help establish a timeline of diabetic wound healing and explore potential changes in vascular density (68).

Furthermore, this study further characterized subpopulations of vascular endothelial cells and identified HE-VasEndo as significantly associated with wound healing. Through intercellular communication analysis, we found that the interactions between B lymphocytes, keratinocytes, M1 macrophages, and HE-VasEndo were weaker in DFU-Non-Healer, highlighting the importance of cellular interactions of endothelial cells in vascular function and angiogenic capacity of the wound (17, 69, 70). Differentiation trajectory analysis showed that HE-VasEndo in DFU-Non-Healer exhibited weaker differentiation potential (71), indicating that the differentiation capacity of endothelial cells is a key factor influencing the healing of DFU wounds. In summary, we have revealed the molecular and functional specificity of vascular endothelial cells in non-healing DFUs at the single-cell level, highlighting the importance of endothelial cell immune-mediated capacity in vascular generation and wound healing. These findings provide new insights for the treatment of diabetic foot ulcers.

## Data availability statement

Publicly available datasets were analyzed in this study. This data can be found here: https://www.ncbi.nlm.nih.gov/geo/query/acc.cgi?acc=GSE165816.

## Ethics statement

The studies involving humans were approved by The First Affiliated Hospital of Sun Yat-Sen University Research Ethics Committee and the Institutional Review Board. The studies were conducted in accordance with the local legislation and institutional requirements. The participants provided their written informed consent to participate in this study.

## Author contributions

YaL: Conceptualization, Data curation, Investigation, Methodology, Software, Writing – original draft, Writing – review & editing. XL: Funding acquisition, Investigation, Methodology, Software, Writing – review & editing. JLZ: Data curation, Investigation, Methodology, Writing – review & editing. FB: Data curation, Investigation, Methodology, Software, Writing – review & editing. YiL: Data curation, Formal Analysis, Investigation, Methodology, Writing – review & editing. JX: Data curation, Formal Analysis, Methodology, Writing – review & editing. PW: Investigation, Validation, Writing – review & editing. YHX: Investigation, Software, Writing – review & editing. JYZ: Supervision, Validation, Investigation, Funding acquisition, Writing – review & editing. SQ: Data curation, Software, Validation, Writing – review & editing. FY: Investigation, Visualization, Writing – review & editing. LC: Funding acquisition, Investigation, Methodology, Project administration, Resources, Supervision, Validation, Visualization, Writing – review & editing. YBX: Funding acquisition, Investigation, Methodology, Project administration, Resources, Supervision, Validation, Visualization, Writing – review & editing.

## Supplementary material

Supplementary Data Sheet 1 Clinical details on skin tissue donors for immunofluorescence staining.

Supplementary Data Sheet 2 Directory of original datasets downloaded from GSE165816 for this research project.

Supplementary Data Sheet 3 The number of different cell types in different groups.

Supplementary Data Sheet 4 Mean, standard deviation and standard error of proportion of different cell types in different groups.

Supplementary Data Sheet 5 Statistical analysis result of significantly different gene expression in pairwise comparison of different groups.

Supplementary Data Sheet 6 The corresponding target genes of HIF-1α and ID1 in TRRUST database.

Supplementary Data Sheet 7 The 43 common intersection pathways enriched in significantly differentially expressed genes and target genes.
