# Correction: Single-cell profiling reveals transcriptomic signatures of vascular endothelial cells in non-healing diabetic foot ulcers

**DOI:** 10.3389/fendo.2025.1714094

**Published:** 2025-11-17

**Authors:** Yangzhou Lu, Xiaogang Liu, Jingling Zhao, Fan Bie, Yiling Liu, Julin Xie, Peng Wang, Junyou Zhu, Yahui Xiong, Shitian Qin, Fan Yang, Lei Chen, Yingbin Xu

**Affiliations:** 1 Department of Burn, Wound Repair & Reconstruction, The First Affiliated Hospital of Sun Yat-Sen University, Guangzhou, Guangdong, China; 2 Guangdong Provincial Engineering Technology Research Center of Burn and Wound Accurate Diagnosis and Treatment Key Technology and Series of Products, Sun Yat-Sen University, Guangzhou, Guangdong, China; 3 Institute of Precision Medicine, The First Affiliated Hospital, Sun Yat-Sen University, Guangzhou, Guangdong, China

**Keywords:** single-cell RNA sequencing, diabetic foot ulcers, non-healing wounds, vascular endothelial cell, immune, inflammation, angiogenesis

There was a mistake in the caption of **Table 1** as published. The corrected caption of **Table 1** appears below.

**Table 1:** Details of antibodies and fluorescent dyes used in immunofluorescence staining.

Name	CD31	CCND1	EN01	HIF1α	SERPINE1	Goat Anti-Rabbit IgG (H+L) HRP	FITC-Tyramide (TSA)	CY3-Tyramide (TSA)	DAPI
Company	Affinity Biosciences	Affinity Biosciences	Affinity Biosciences	Affinity Biosciences	Affinity Biosciences	Affinity Biosciences	Servicebio	Servicebio	Abcam
Catalog Number	AF6191	AF0931	DF6191	AF1009	AF5176	S0001	G1222	G1223	Ab228549
Dilution	1:200	1:200	1:100	1:200	1:100	1:200	1:500	1:500	1:10000

“Details of Antibodies and Fluorescent Dyes Used in Immunofluorescence Staining.”

The original version of this article has been updated.

