# Supplementary figures and images for: Single-cell profiling reveals transcriptomic signatures of vascular endothelial cells in non-healing diabetic foot ulcers
Source: Front Endocrinol (Lausanne). 2023 Dec 1;14:1275612. doi: 10.3389/fendo.2023.1275612 (PMC10722230; doi:10.3389/fendo.2023.1275612)

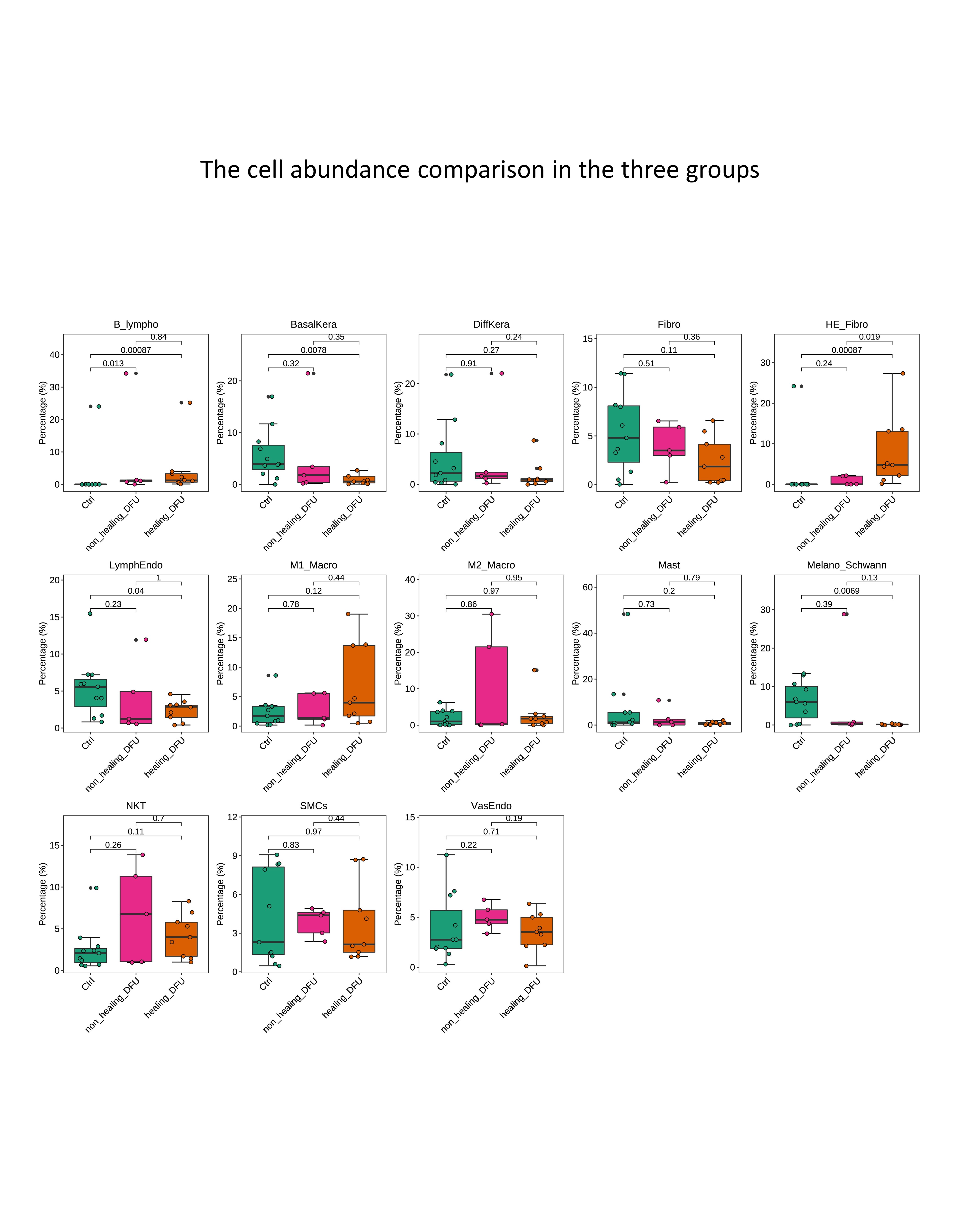

Supplement: Supplementary Data Sheet 1 — Clinical details on skin tissue donors for immunofluorescence staining. [file DataSheet_1.zip › Data sheet 1/Supplementary figure/Supplementary figure 1.jpg]

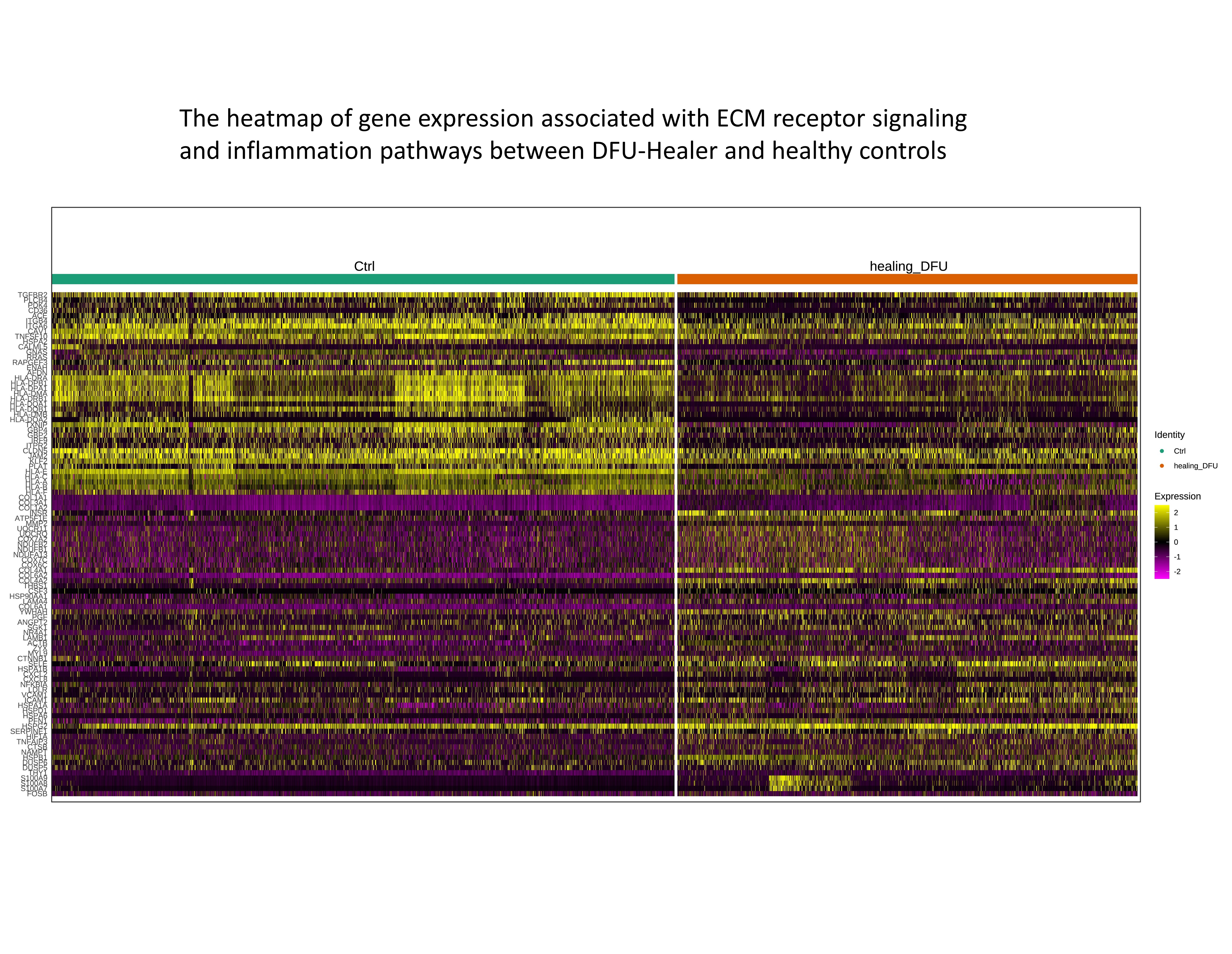

Supplement: Supplementary Data Sheet 1 — Clinical details on skin tissue donors for immunofluorescence staining. [file DataSheet_1.zip › Data sheet 1/Supplementary figure/Supplementary figure 2.jpg]

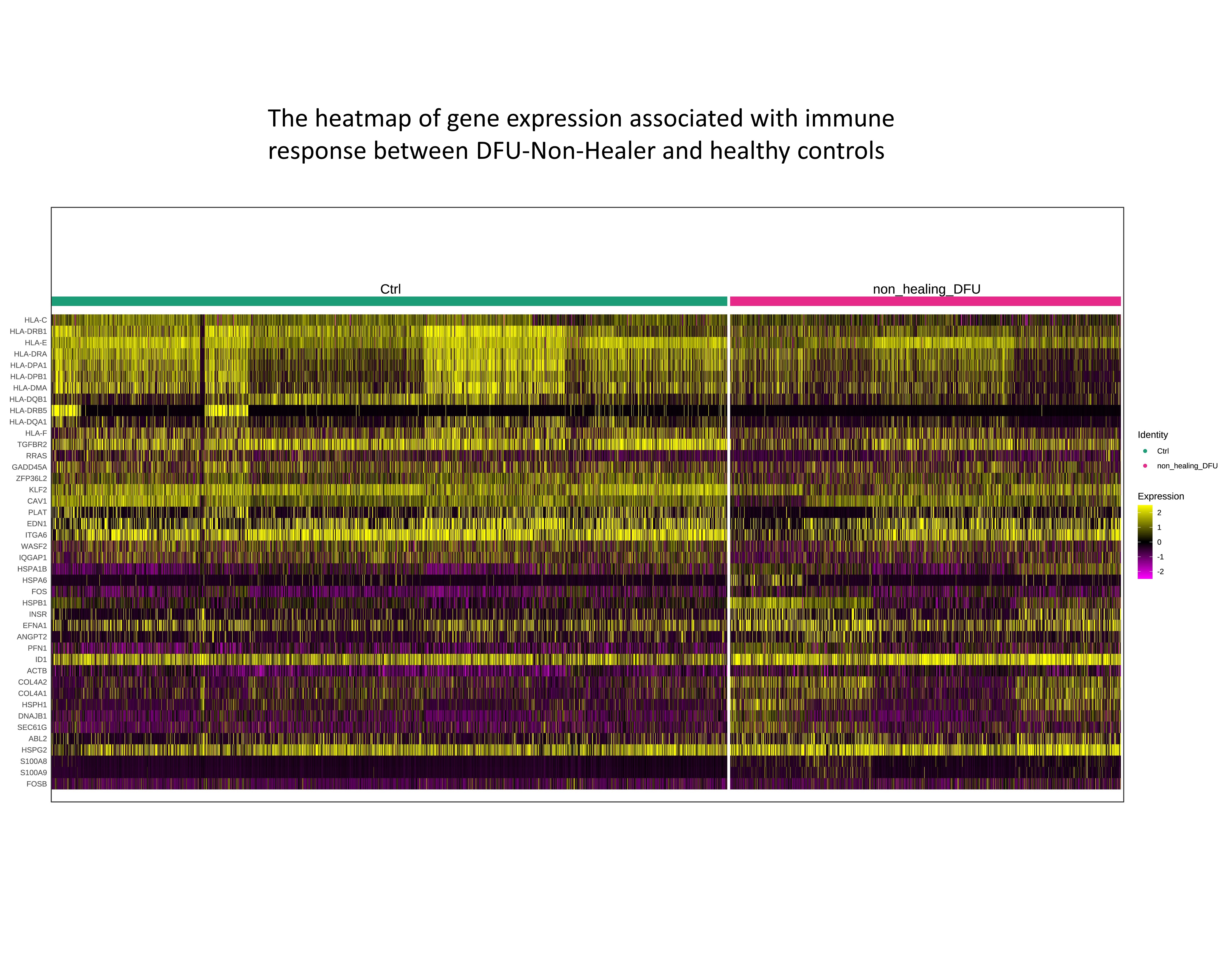

Supplement: Supplementary Data Sheet 1 — Clinical details on skin tissue donors for immunofluorescence staining. [file DataSheet_1.zip › Data sheet 1/Supplementary figure/Supplementary figure 3.jpg]

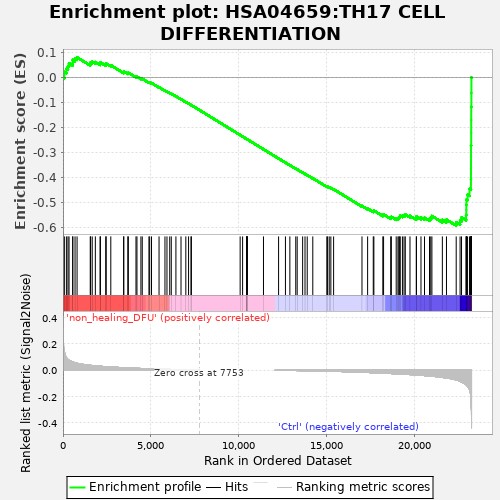

Supplement: Supplementary Data Sheet 1 — Clinical details on skin tissue donors for immunofluorescence staining. [file DataSheet_1.zip › Data sheet 1/Supplementary figure/Supplementary figure 4.jpg]

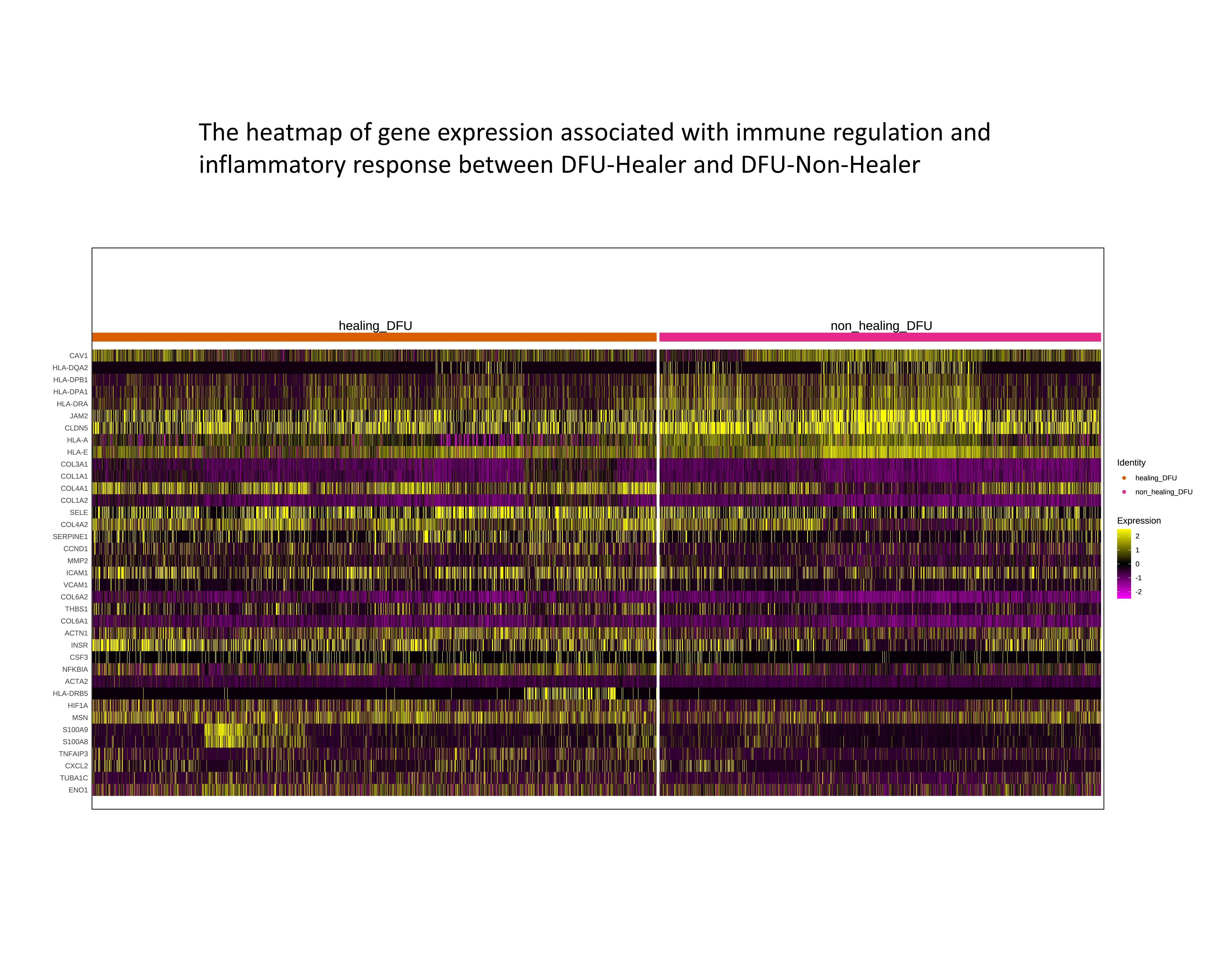

Supplement: Supplementary Data Sheet 1 — Clinical details on skin tissue donors for immunofluorescence staining. [file DataSheet_1.zip › Data sheet 1/Supplementary figure/Supplementary figure 5.jpg]

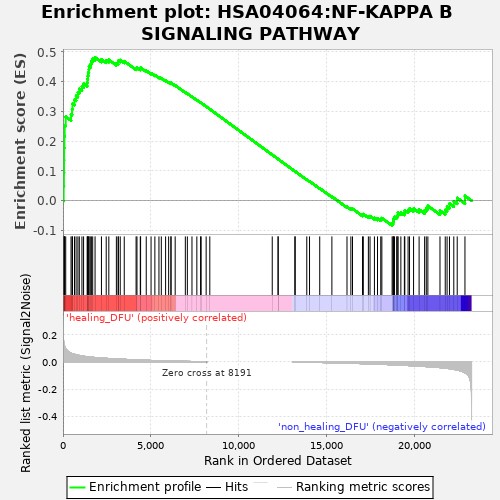

Supplement: Supplementary Data Sheet 1 — Clinical details on skin tissue donors for immunofluorescence staining. [file DataSheet_1.zip › Data sheet 1/Supplementary figure/Supplementary figure 6/Supplementary figure 6-1 VasEndo.GSEA.healing_DFU to non_healing_DFU.hsa04064.plot.jpg]

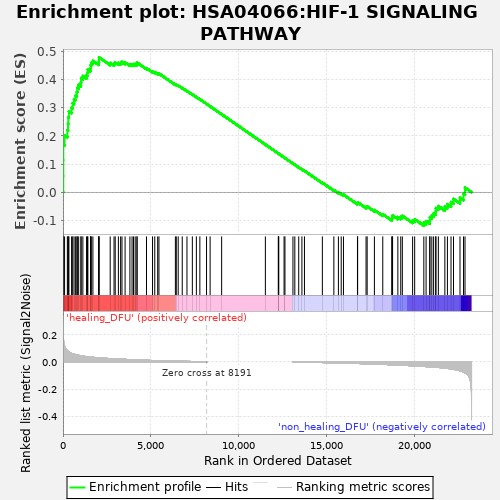

Supplement: Supplementary Data Sheet 1 — Clinical details on skin tissue donors for immunofluorescence staining. [file DataSheet_1.zip › Data sheet 1/Supplementary figure/Supplementary figure 6/Supplementary figure 6-2 VasEndo.GSEA.healing_DFU to non_healing_DFU.hsa04066.plot.jpg]

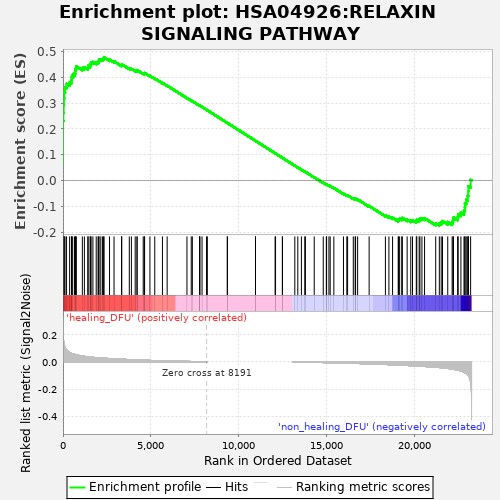

Supplement: Supplementary Data Sheet 1 — Clinical details on skin tissue donors for immunofluorescence staining. [file DataSheet_1.zip › Data sheet 1/Supplementary figure/Supplementary figure 6/Supplementary figure 6-3 VasEndo.GSEA.healing_DFU to non_healing_DFU.hsa04926.plot.jpg]

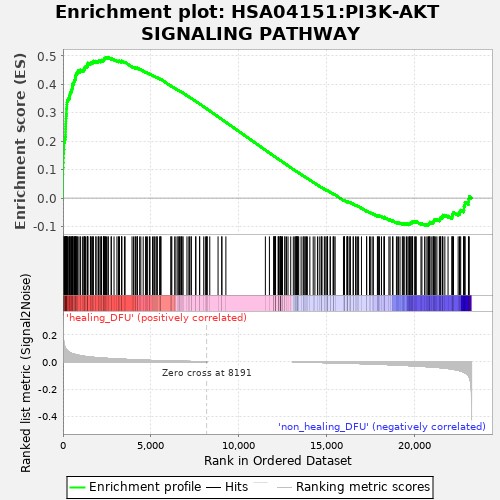

Supplement: Supplementary Data Sheet 1 — Clinical details on skin tissue donors for immunofluorescence staining. [file DataSheet_1.zip › Data sheet 1/Supplementary figure/Supplementary figure 6/Supplementary figure 6-4 VasEndo.GSEA.healing_DFU to non_healing_DFU.hsa04151.plot.jpg]

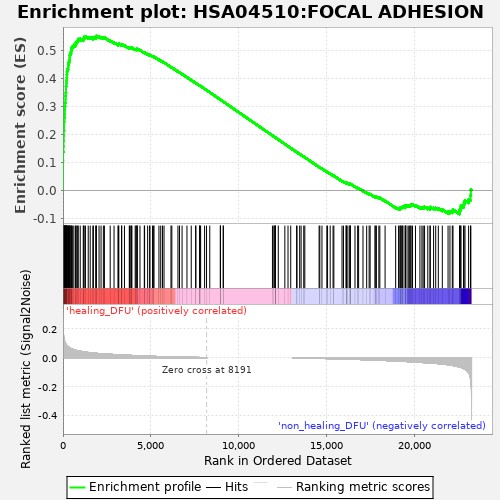

Supplement: Supplementary Data Sheet 1 — Clinical details on skin tissue donors for immunofluorescence staining. [file DataSheet_1.zip › Data sheet 1/Supplementary figure/Supplementary figure 6/Supplementary figure 6-5 VasEndo.GSEA.healing_DFU to non_healing_DFU.hsa04510.plot.jpg]

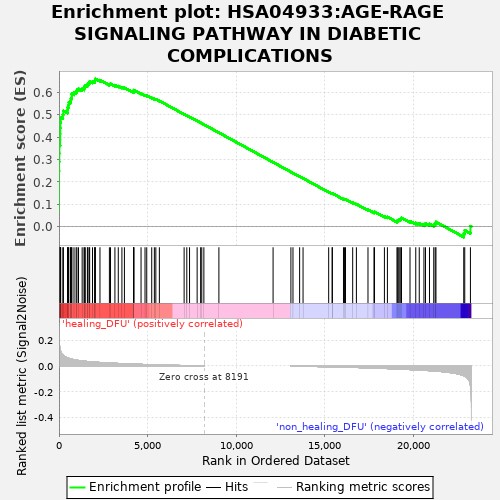

Supplement: Supplementary Data Sheet 1 — Clinical details on skin tissue donors for immunofluorescence staining. [file DataSheet_1.zip › Data sheet 1/Supplementary figure/Supplementary figure 6/Supplementary figure 6-6 VasEndo.GSEA.healing_DFU to non_healing_DFU.hsa04933.plot.jpg]

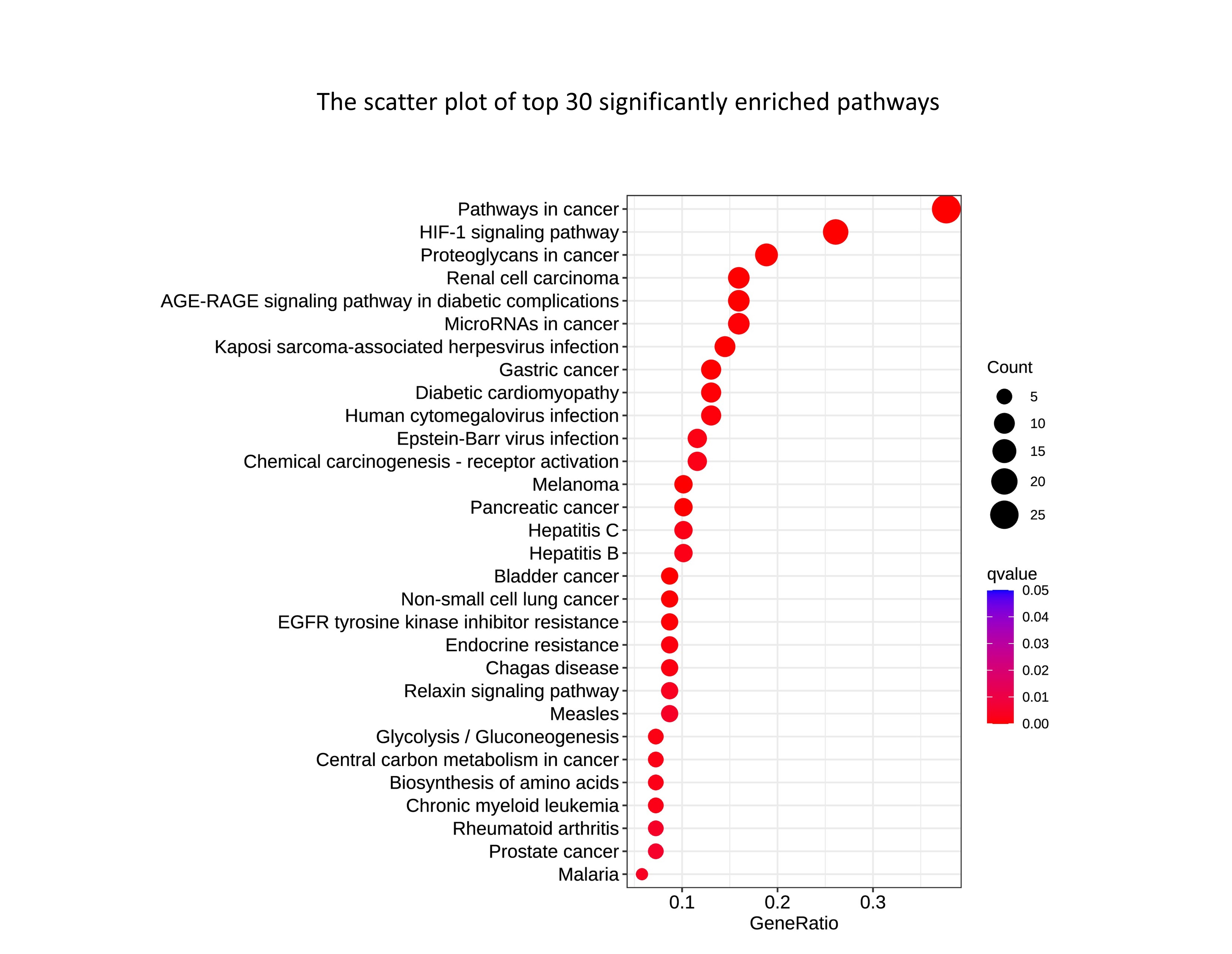

Supplement: Supplementary Data Sheet 1 — Clinical details on skin tissue donors for immunofluorescence staining. [file DataSheet_1.zip › Data sheet 1/Supplementary figure/Supplementary figure 7.jpg]

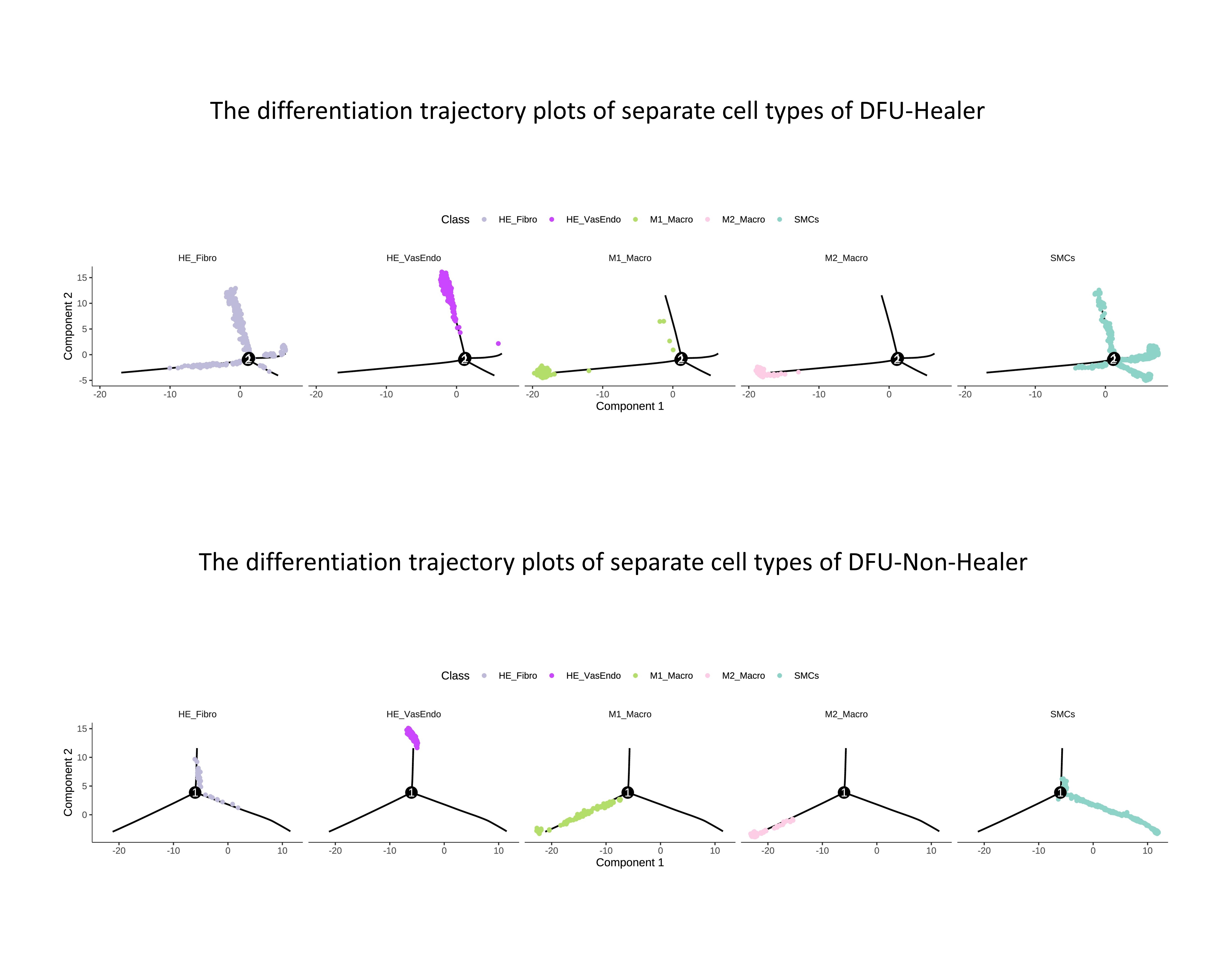

Supplement: Supplementary Data Sheet 1 — Clinical details on skin tissue donors for immunofluorescence staining. [file DataSheet_1.zip › Data sheet 1/Supplementary figure/Supplementary figure 8.jpg]
